# Genome Assembly of Salicaceae *Populus deltoides* (Eastern Cottonwood) *I-69* Based on Nanopore Sequencing and Hi-C Technologies

**DOI:** 10.1093/jhered/esab010

**Published:** 2021-03-17

**Authors:** Shengjun Bai, Hainan Wu, Jinpeng Zhang, Zhiliang Pan, Wei Zhao, Zhiting Li, Chunfa Tong

**Affiliations:** Co-Innovation Center for Sustainable Forestry in Southern China, College of Forestry, Nanjing Forestry University, Nanjing, China

**Keywords:** *Populus deltoides*, genome assembly, Nanopore sequencing, chromosome conformation capture technology

## Abstract

*Populus deltoides* has important ecological and economic values, widely used in poplar breeding programs due to its superior characteristics such as rapid growth and resistance to disease. Although the genome sequence of *P. deltoides WV94* is available, the assembly is fragmented. Here, we reported an improved chromosome-level assembly of the *P. deltoides* cultivar *I-69* by combining Nanopore sequencing and chromosome conformation capture (Hi-C) technologies. The assembly was 429.3 Mb in size and contained 657 contigs with a contig N50 length of 2.62 Mb. Hi-C scaffolding of the contigs generated 19 chromosome-level sequences, which covered 97.4% (418 Mb) of the total assembly size. Moreover, repetitive sequences annotation showed that 39.28% of the *P. deltoides* genome was composed of interspersed elements, including retroelements (23.66%), DNA transposons (6.83%), and unclassified elements (8.79%). We also identified a total of 44 362 protein-coding genes in the current *P. deltoides* assembly. Compared with the previous genome assembly of *P. deltoides WV94*, the current assembly had some significantly improved qualities: the contig N50 increased 3.5-fold and the proportion of gaps decreased from 3.2% to 0.08%. This high-quality, well-annotated genome assembly provides a reliable genomic resource for identifying genome variants among individuals, mining candidate genes that control growth and wood quality traits, and facilitating further application of genomics-assisted breeding in populations related to *P. deltoides*.

*Populus deltoides* is one of the approximately 30 species in the genus *Populus* (Salicaceae), which can be classified into 6 sections, including Abaso, Aigeiros, Leucoides, Populus, Tacamahaca, and Turanga (Eckenwalder 1996). *Populus deltoides* belongs to the section Aigeiros and is naturally distributed from the southeastern United States to southern Canada (Fahrenkrog et al. 2017). It plays an important role in forest breeding research and its genetic resources have become the main gene donors of poplar cultivars (Chen et al. 2020). One of the most important cultivars generated from this species, namely *I-69*, has the superior characteristics of rapid growth and resistance to *Marssonina* leaf spot disease, which usually causes significant economic and ecological losses in poplar plantations in most regions of China (Han et al. 2000; Zhu et al. 2012). To accelerate the domestication of *P. deltoides* for improving the wood quality and yield to meet the growing demand in industry, a variety of genetic and genomic resources need to be developed, especially the high-quality genome sequence (Fang et al. 2018). Furthermore, an accurate genome sequence would be beneficial to performing comparative genomics and studying the evolution of the species.

In forest trees, poplar genomes are relatively small, but they are highly heterozygous, and possess abundant repetitive sequences (Michael and VanBuren 2020). In recent years, the genomes of several poplar species have been sequenced, assembled, and annotated, including *Populus trichocarpa* (Tuskan et al. 2006), *Populus pruinosa* (Yang et al. 2017), *Populus alba* (Liu et al. 2019), *Populus euphratica* (Zhang et al. 2020), and *Populus simonii* (Wu et al. 2020). Although the genome sequence of *P. deltoides WV94* is available in the Phytozome database (http://phytozome.jgi.doe.gov), it contains many gaps (3.2%) and has a lot of sequences that were unanchored into chromosomes, thereby impeding downstream analysis. Therefore, there is an active demand to improve the current assembly of *P. deltoides* genome. Recent advances in Nanopore sequencing and chromosome conformation capture (Hi-C) technologies play an important role in improving plant genomes at the chromosome level (Giani et al. 2020). Nanopore sequencing exhibits many advantageous qualities, including high sequencing throughput, low cost, and extremely long-read lengths that can span repetitive regions (Cali et al. 2019). Moreover, Hi-C technology has emerged as a robust tool to reconstruct genomes at the chromosome level, providing long-range information about the grouping and linear organization of sequences along entire chromosomes (Burton et al. 2013). Recently, several high-quality plant genomes had been obtained by using Nanopore sequencing and Hi-C technologies, including *Eriobotrya japonica* (Jiang et al. 2020), *Aquilaria sinensis* (Ding et al. 2020), and *Asparagus setaceus* (Li et al. 2020).

In this study, we performed the de novo genome assembly of the *P. deltoides* cultivar *I-69* using Nanopore sequencing and Hi-C technologies. The *P. deltoides I-69* was originally collected from a natural population in Illinois, United States, and selected as a cultivar in Italy in the 1950s. Later on, it was introduced to China in 1972 and greatly improved industrial timber production, agroforestry and ecoremediation since then (Zhang et al. 2009). As a female parent, the *P. deltoides I-69* was crossed with *P. simonii L-3* to generate an F_1_ hybrid population for constructing genetic linkage map and locating quantitative trait loci (QTL) in *Populus* (Mousavi et al. 2016; Tong et al. 2016). Although we successfully obtained the genome sequence of the male parent of *P. simonii* in a previous study (Wu et al. 2020), the lack of high-quality genomic information of the female parent *P. deltoides I-69* was a barrier to develop new cultivars in the F_1_ hybrid population. Therefore, we conducted the whole genome sequencing and performed de novo genome assembly of the female parent, resulting in an improved genome sequence of *P. deltoides*. The high-quality assembly of the *P. deltoides* genome provided a valuable resource for poplar breeding and genetic improvement, as well as comparative genomics analysis with related species.

## Methods

### Biological Materials

A single cutting of the *P. deltoides* cultivar *I-69* was collected from the Siyang Forest Farm in Siyang country, Jiangsu Province, China and planted at the Xiashu Forest Farm of Nanjing Forestry University, Jurong, Jingsu Province, China in 2012 (Mousavi et al. 2016). Fresh leaves were collected from the clonal tree, immediately transferred to liquid nitrogen, and stored until DNA extraction.

### DNA Sequencing and Genome Assembly

Genomic DNA was extracted using the Qiagen DNeasy Blood and Tissue kit and evaluated on a 0.75% agarose gel. The concentration and purity of the genomic DNA were determined by Nanodrop (OD260/280) and Qubit (Thermo Fisher Scientific). To generate Nanopore long reads, ~15 µg of genomic DNA was size-selected (>20 Kb) with a Blue Pippin System (Sage Science). According to *1D Genomic DNA by Ligation* protocol (Oxford Nanopore Technologies, ONT), ONT SQK-LSK108 library preparation kit was used to construct PCR-free libraries. Nextomics (Nextomic Biosciences Technologies Corporation, Wuhan, China) and Biozeron (Shanghai Biozeron Biotechnology Corporation, Shanghai, China) performed the Nanopore sequencing of prepared libraries on 2 flow-cells using the GrildION X5 sequencer according to the manufacturer’s instructions (Oxford Nanopore, Oxford, UK). The raw signal data were base-called using ONT MinKNOW software (v1.4.2), and the statistics of the generating Nanopore sequencing data were calculated using Nanostat (v0.8.1) (De Coster et al. 2018).

In addition, using the same individual tree described above, we sequenced the whole genome of *P. deltoides* by the Illumina HiSeq 2000 sequencing platform at BMK (Biomarker Technologies Corporation, Beijing, China). The detailed procedure used for whole-genome sequencing was described in Mousavi et al. (2016). The paired-end (PE) reads data are available from the NCBI SRA database under the accession number SRP071167. For the purpose of correcting the contigs assembled with the Nanopore sequencing data, these whole-genome sequencing data were processed to filter the low-quality reads using the NGS QC Toolkit (v2.3.3) (Patel and Jain 2012) with default parameters to obtain high-quality reads.

To assist gene prediction, RNA-sequencing (RNA-seq) data were generated from samples of leaf and stem tissue. Total RNA was isolated using TRIzol Reagent (Invitrogen). RNA libraries were prepared using the TruSeq RNA library Preparation Kit (Illumina), and then sequenced on the Illumina HiSeq 2000 platform with PE mode at Beijing Genomics Institute (BGI). The generating RNA-seq data were also filtered using the NGS QC Toolkit (v2.3.3) with default parameters (Patel and Jain 2012).

The Hi-C library was constructed and sequenced by Frasergen (Wuhan Frasergen Biotechnology Corporation, Wuhan, China) following the standard procedures. Briefly, nuclear chromatin was cross-linked by 1% formaldehyde at room temperature for 30 min. The cross-linked DNA was extracted and then digested with *Mbo*I restriction enzyme at 37 °C for 4 h, end-labeled with biotin, and ligated using T4 DNA ligase. The cross-links were reversed by overnight incubation at 65 °C, and the circular DNA was sheared into 300–500 bp fragments. After end repairing, the DNA fragments were purified and sequenced on Illumina HiSeq X Ten platform with PE mode.

The software packages used in the genome assembly and annotation were summarized in Table 1. Oxford Nanopore sequencing data were employed for genome assembly by using the software Canu (v1.9) with default parameters (Koren et al. 2017). To improve the base accuracy, the assembled genome sequence was polished in 3 steps. First, the Nanopore reads were mapped back to the assembled contigs with minimap2 (v2.12) (Li 2018), and then these contigs were polished 3 rounds with Racon (v1.4.3) with default parameters (Vaser et al. 2017). Second, one round of Medaka (v1.4.3) (https://github.com/nanoporetech/medaka) polishing was performed with parameter “-m r941_min_fast_g303” using the Nanopore reads. Third, high-quality Illumina short reads were mapped to the Medaka consensus sequence using BWA-MEM (v0.7.17) (Li 2013), and then subjected to 3 rounds of polishing with NextPolish (v1.1.0) (Hu et al. 2020). Additionally, considering the high heterozygosity of forest tree genome could hinder downstream analysis, we aligned the Nanopore reads to the polished contigs using Minimap2 (v2.12) (Li 2018). Based on this alignment, Purge Haplotigs (v1.1.1) (Roach et al. 2018) was used to identify and remove redundant heterozygous sequences, resulting in the draft genome of the *P. deltoides I-69*.

**Table 1.** Software used for genome assembly and annotation pipeline in the current study

	Software	Version
Genome assembly pipeline		
De novo assembly	Canu	v1.9
Contig polishing	Racon	v1.4.3
	Medaka	v1.4.3
	NextPolish	v1.1.0
Remove heterozygous sequences	Minimap2	v2.12
	Purge Haplotigs	v1.1.1
Hi-C contact map generation	Juicer	v1.7.6
Hi-C scaffolding	3D-DNA	v180114
Manual assembly inspection	Juicebox Assembly Tools	v1.11.8
Genome evaluation		
Assembly completeness	BUSCO	v4.0.1
Read mapping	BWA-MEM	v0.7.17
SNP calling	Sambamba	v0.7.1
	GATK	v4.1.8
Repeat annotation		
Construct a de novo repeat library	RepeatModeler	v2.0.1
Repeat assessment	RepeatMasker	v4.1.0
Gene prediction		
De novo transcript assembly	Trinity	v2.1.1
Transcript evidence	PASA	v2.3.3
Homology prediction	tBlastn	v2.2.31
	Exonerate	v2.4.0
Ab initio prediction	SNAP	v2006-07-28
	GeneMark-ES	v4.59
	AUGUSTUS	v3.3.3
Integrate all gene structures	EVM	v1.1.1
Update final gene sets	PASA	V2.3.3

To obtain the chromosome-level genome, we aligned the Hi-C reads to the draft genome and computed the Hi-C contact frequency between genomic loci using the Juicer pipeline (v1.7.6) (Durand et al. 2016). Afterwards, the contigs were corrected for mis-joins, ordered, oriented, and anchored into a candidate chromosome-length assembly using the 3D-DNA (v180114) with default parameters (Dudchenko et al. 2017). The candidate assembly was visualized with the Juicebox Assembly Tools (JBAT v1.11.8) (Durand et al. 2016). Then the misassembled contigs or the mis-joined scaffolds were manually detected and corrected based on the appearance of a bright band of elevated contact frequency along the diagonal of the Hi-C heatmap (Dudchenko et al. 2018). After JBAT review, we used the “run-asm-pipeline-post-review.sh” in 3D-DNA to generate the adjusted assembly, and then the scaffolds shorter than 2 Kb were removed.

### Genome Evaluation

To assess the quality of the genome assembly, we mapped the high-quality Illumina short reads back to the final assembled genome using BWA-MEM (v0.7.17) (Li 2013). Then the duplicated reads were marked using the “markdup” function of Sambamba (v0.7.1) (Tarasov et al. 2015), and variant calling was performed to evaluate the accuracy of the genome at the single-base level using GATK HaplotypeCaller (v4.1.8) (DePristo et al. 2011). We further performed Benchmarking Universal Single-Copy Orthologs (BUSCO v4.0.1) analysis to evaluate the completeness of the *P. deltoides I-69* assembly with embryophyta_odb10 database (Simao et al. 2015).

### Repetitive Sequences Annotation

Plant genomes usually consist of large numbers of repeat elements that have been demonstrated to have structural and functional roles (Biscotti et al. 2015). Repetitive sequences were annotated using RepeatMasker (v4.1.0) (Tarailo-Graovac and Chen 2009) based on a combined library generated by de novo-based and homology-based approaches. We used the software RepeatModeler (v2.0.1) (Tarailo-Graovac and Chen 2009) to construct a de novo repeat library with the assembled genome sequence as input. Then combined with the known repeat library from Repbase (Bao et al. 2015), RepeatMasker was used to perform a homology-based repeat search. To ensure the integrity of genes in the subsequent analyses, low complexity or simple repeats were not masked because some of these sequences could be within genes (Xing et al. 2019).

### Gene Prediction

The repeat-masked genome was used for annotating gene models by combining the methods of homology-based, RNA-Seq-assisted, and ab initio predictions. With the homology-based prediction approach, protein sequences from the genome annotations of *P. trichocarpa* (Tuskan et al. 2006), *P. simonii* (Wu et al. 2020), *Salix suchowensis* (Wei et al. 2020), *P. euphratica* (Zhang et al. 2020), and *P. deltiodes WV94* (http://phytozome.jgi.doe.gov) were collected and aligned against the genome using tBlastn (v2.2.31) (Altschul et al. 1997). The blast hits were refined for exact intron and exon positions using Exonerate (v2.4.0) (Slater and Birney 2005). For RNA-seq-assisted prediction, high-quality Illumina RNA-Seq reads were de novo assembled into transcripts using Trinity (v2.1.1) with default parameters (Grabherr et al. 2011), and then these transcripts were aligned to the genome using PASA (v2.3.3) (Haas et al. 2003) to determine the potential gene models. For ab initio prediction, the following 3 ab initio prediction software packages were used: AUGUSTUS (v3.3.3) (Stanke et al. 2006), SNAP (v2006-07-28) (Korf 2004), and GeneMark (v4.59) (Ter-Hovhannisyan et al. 2008). The prediction results obtained from the above 3 methods were integrated by EVidenceModeler (v1.1.1) (Haas et al. 2008). Finally, PASA was used once again, in conjunction with transcripts, to update the untranslated regions and alternate splicing information of the predicted genes. The quality of the gene annotation was assessed using BUSCO (v4.0.1) (Simao et al. 2015) with the parameters: “-m proteins -l embryophyta_odb10.”

## Results

### Genome Assembly

A total of 44.4 Gb long-read data was obtained from the Oxford Nanopore sequencing platform, covering ~100× of the *P. deltoides* genome, with an average sequence length of 16 Kb and N50 of 25 Kb (Supplementary Figure 1). In addition to the long-read data, the data of Illumina short reads, Hi-C reads, and RNA-seq reads were also obtained and summarized in Table 2. De novo assembly was performed using the Canu software with the Nanopore sequencing data, and further polished for base accuracy in 3 steps (see Methods). As a result, the primary assembly of the *P. deltoides* consisted of 2186 contigs, amounting to 545 Mb with a contig N50 length of 1.83 Mb, and the length of longest contigs reached 16.8 Mb. After removing the potential heterozygous sequences in the primary genome (Supplementary Figure 2), the assembly size reduced to 429 Mb, with the number of contigs reduced to 657 and length of contig N50 increased to 2.62 Mb (Table 3).

**Table 2.** Summary of sequencing data for the genome assembly and annotation of *Populus deltoides I-69*

Data type	Number of reads	Total bases(bp)	Coverage
HQ Illumina sequencing	235 343 502	23 769 512 714	55×
Nanopore sequencing	2 752 090	44 352 852 004	100×
Hi-C sequencing	340 036 044	51 005 406 600	118×
HQ RNA-seq of stem	49 765 508	7 464 826 200	17×
HQ RNA-seq of leaf	47 295 142	4 256 562 780	10×

HQ, high-quality.

**Table 3.** Comparison between the assemblies of *Populus deltoides WV94* and *P. deltoides I-69* genomes

Assembly statistics	*P. deltoides WV94*	*P. deltoides I-69*
Assembly size (Mb)	446.8	429.3
Total number of contigs	NA	657
Longest contigs (Mb)	NA	16.8
Contig N50 (Mb)	0.59	2.62
Scaffold N50 (Mb)	21.7	21.5
Total number of scaffolds	1375	934
19 chromosomes (%)	90.2	97.4
Gaps (%)	3.2	0.08
GC content (%)	32.32	33.38
Complete BUSCOs (%)	97.7	98.2

NA, not applicable.

**Figure 1.**
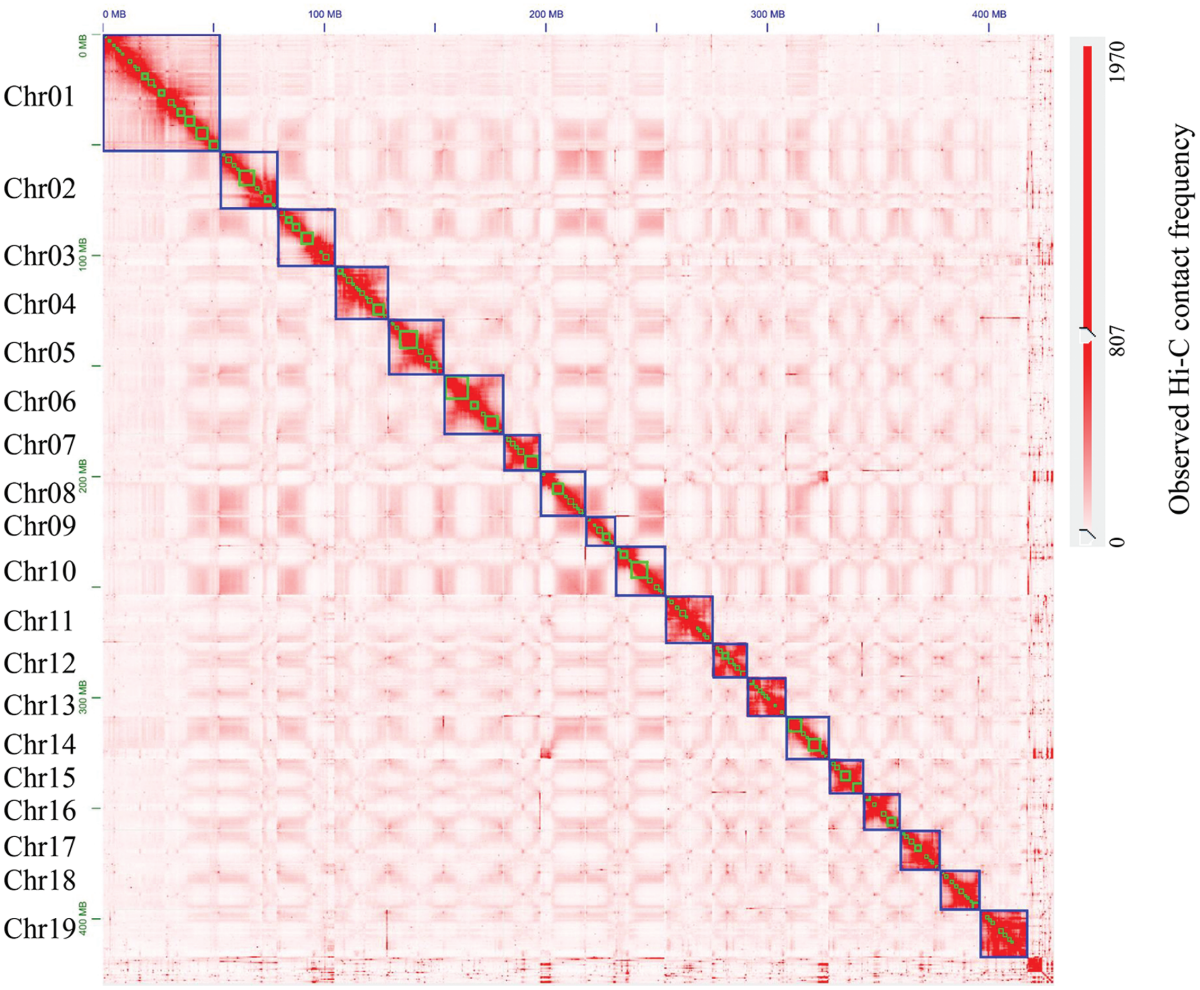
The genome-wide Hi-C interaction heatmap of 19 chromosomes in *Populus deltoides*. Each chromosome is framed with a bigger block, and each contig is framed with a smaller block. Heatmap shows Hi-C interactions under the resolution of 500 Kb. Deeper color indicates higher contact frequency.

To construct the chromosome-level genome, a total of 51.04 Gb Hi-C reads were generated. Before scaffolding the assembled contigs, we assessed the quality of Hi-C data, of which 99.18% were mapped to the draft genome and 46.9% were uniquely mapped. Based on Hi-C linking information (Figure 1), 421 contigs were identified as containing assembly errors and broken into 1716 contigs by using the 3D-DNA software. Then, 3D-DNA assigned 776 contigs with a total size of 418 Mb (97.4%) into 19 groups, perfectly matching the karyotype of *Populus*. Each group represented a chromosome-level assembly of which the order number was determined by aligning its sequence to the genome sequences of the *P. deltoides W94* using Minimap2 (v2.12) (Li 2018). The length of the largest chromosome was 53.17 Mb, while the smallest one was 13.45 Mb. The scaffold N50 of the chromosome-level genome assembly reached 21.51 Mb (Supplementary Table 1).

### Genome Evaluation

Based on the alignment of the high-quality Illumina short reads (Table 2) to the genome, the high mapped rate (98.82%) and coverage rate (96.9%) indicated a high consistency between the reads and the genome assembly. Through variant calling, we identified a total of 2 744 622 SNPs (0.64% of the genome), of which 63 341 SNPs (0.0147% of the genome) belonged to homozygous SNPs, indicating a high accuracy of genome assembly at the single-base level (Liang et al. 2020). The completeness of the genome assembly was evaluated using BUSCO with embryophyta_odb10 database. Of the 1614 plant-specific orthologs, 1585 (98.2%) were identified to be complete BUSCO genes, with 0.3% being partial BUSCOs identified and only 1.5% missed. Overall, all the results suggested that the quality of the assembly was high with respect to the base level accuracy and the completeness of the assembly. In addition, the alignment of our *P. deltoides I-69* genome to the *P. deltoides W94* genome with the MUMmer software (Kurtz et al. 2004) showed that there was a high degree of synteny between the 2 genome sequences (Krzywinski et al. 2009) (Supplementary Figure 3). Moreover, the differences in chromosome sizes among the genomes available in *Populus* were summarized in Supplementary Table 2.

### Repetitive Sequences Annotation

Repetitive sequences analysis of the assembly showed that the interspersed repeats comprised approximately 169 Mb (39.28%) of the *P. deltoides* genome, including retroelements (23.66%), DNA transposons (6.83%), and unclassified elements (8.79%) (Supplementary Table 3). This result was similar to the genomes of *P. trichocarpa* (44.06%) and *P. alba* (41.08%) (Liu et al. 2019).

### Gene Prediction

**Figure 2.**
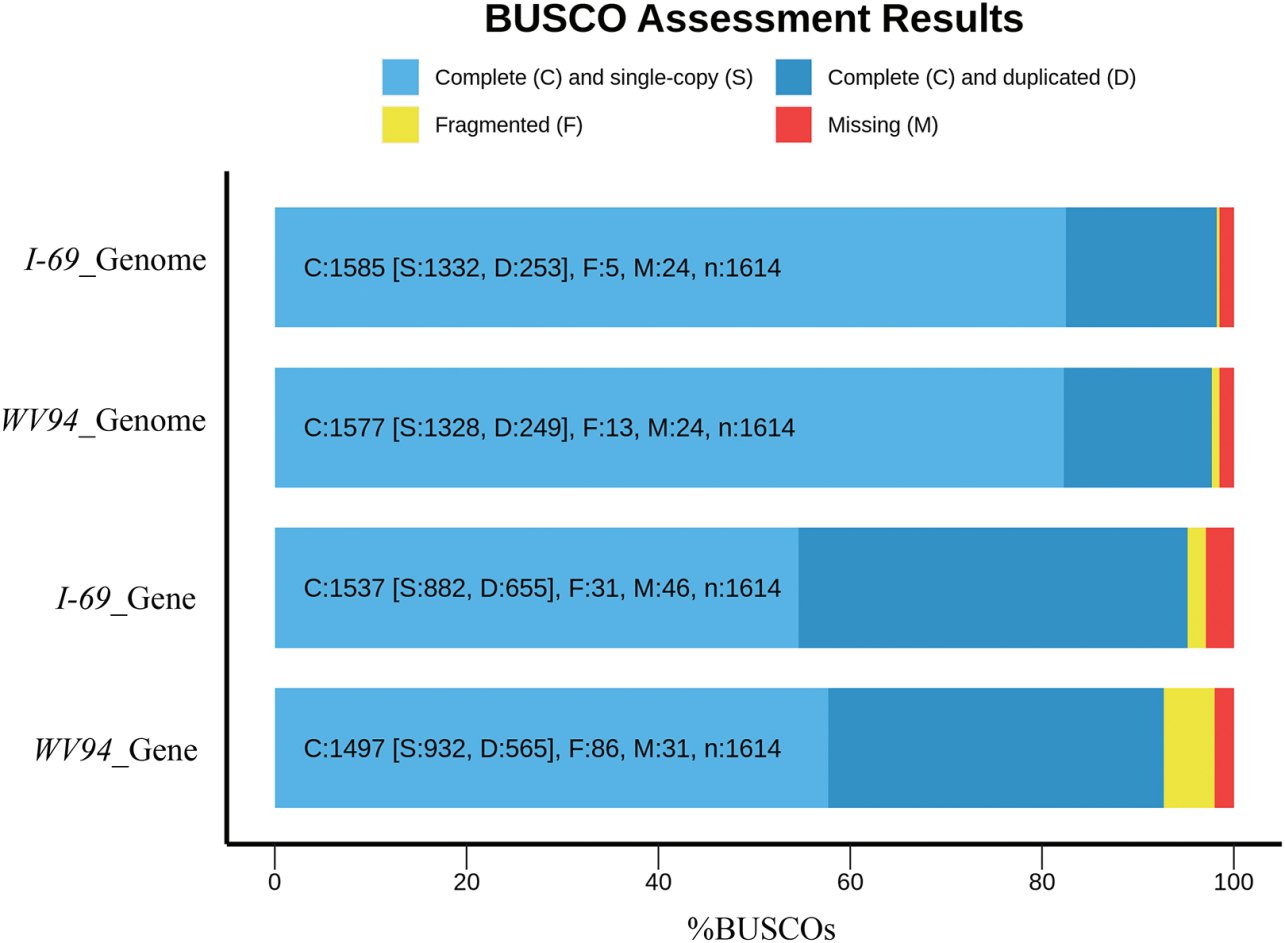
Comparison of the BUSCO analysis between genome assemblies and gene annotations of *Populus deltoides I-69* and *P. deltoides WV94*.

The Trinity assembly of the RNA-seq data contained 117 189 transcripts with a contig N50 of 2134 bp. Combining the ab initio, homology-based, and RNA-seq-assisted predictions, a total of 32 245 genes encoding 44 362 proteins were predicted in the *P. deltoides* genome. The average CDS length was 1258bp, with the average number of exons was 5.6 for a single CDS (Supplementary Table 4). With the obvious improvement in the contiguity of the *P. deltoides I-69* genome, BUSCO analysis showed that the *P. deltoides I-69* had more complete BUSCO genes identified (95.2%) than *P. deltoides WV94* (92.7%) in the protein-coding genes (Figure 2). Furthermore, the software OrthoVenn2 was used to identify orthologous genes among the genomes of *P. deltoides*, *P. euphratica*, *P. trichocarpa*, *P. simonii*, and *S. suchowensis* (Lee et al. 2020). These species shared a total of 14 446 orthologous groups, and 986 unique orthologous groups for *P. deltoides* were identified (Supplementary Figure 4).

## Discussion

The genome sequence of the *P. deltoides I-69* was assembled by integrating Nanopore sequencing and Hi-C technologies, exhibiting some improvements over the genome assembly of *P. deltoides WV94* which was assembled using PacBio sequencing technology and a genetic linkage map (http://phytozome.jgi.doe.gov). First, the contig N50 length of our assembly reached 2.62 Mb, a 3.5-fold improvement over that of *P. deltoides WV94* (0.59 Mb), possibly due to the Nanopore reads generally being longer than the SMRT-seq reads (Rice and Green 2019). Second, although *P. deltoides WV94* genome had its contigs assigned into chromosomes based on a genetic linkage map, there remained 1356 scaffolds with total size of 43.6 Mb unanchored, accounting for 9.8% of the assembly. By applying Hi-C technology, we accurately constructed 19 chromosomes with total size of 418 Mb accounting for 97.4% of the assembly size, only leaving about 12 Mb sequences unanchored into chromosomes. Third, the genome completeness analysis showed that more completely conserved genes (98.2%) were found in the *P. deltoides I-69* than *P. deltoides WV94* (97.5%) (Figure 2). These aspects demonstrated that our newly assembled genome achieved a higher level of continuity and quality, suggesting that it can be a good alternative resource for *Populus*. High heterozygosity is a common feature in forest tree genomes, and the software Canu assembles heterozygous regions into separate contigs when a pair of allelic sequences exceeds a certain threshold of nucleotide diversity, rather than the expected single haplotype-fused contig (Roach et al. 2018). Thus, the overall assembly size resulted from Canu will be larger than the haploid genome size, making it difficult to scaffold contigs and predict genes downstream (Guan et al. 2020). To solve such a problem, we applied the Purge Haplotigs software to remove the heterozygous sequences of the *P. deltoides I-69* genome in this study (Roach et al. 2018). Before the analysis with Purge Haplotigs, the assembly size of the *P. deltoides I-69* genome was 545 Mb, substantially exceeding the size of the existed *P. deltoides WV94* genome (447 Mb). After the removing process, the assembly size of the *P. deltoides I-69* reduced to 429 Mb, and the contig N50 increased from 1.8 to 2.62 Mb. Meanwhile, BUSCO analysis showed that the proportion of complete BUSCO genes was also increased from 96.6% to 98.2%. The most obvious effect was that the duplicated BUSCO proportion decreased from 30.9% to 15.7%, and the proportion of the complete and single-copy BUSCOs increased from 65.7% to 82.5%, suggesting that the heterozygosity of the *P. deltoides I-69* genome reached a lower level (Ran et al. 2019).

With the advances in genomic assembly technologies, Hi-C technology was able to identify more complete sequences at the chromosome level, but it still contained some assembly errors during scaffolding (Giani et al. 2020). For instance, the folding of chromatin in the topologically associated domain could result in higher interaction frequencies of distant regions along chromosome, which will potentially cause misestimation of the distance between 2 adjacent regions along chromosome, leading to some artificial inversions and mis-joined scaffolds during assembly (Oddes et al. 2018; Xu and Dixon 2020). These errors can be better corrected by resorting a dense genetic map, so that several species had applied genetic linkage maps to refine assemblies at present (Maroso et al. 2018; Gaur et al. 2020; Shi et al. 2020). Therefore, efforts could be made to construct a high-quality, high-density linkage map of *P. deltoides* for further refining the *P. deltoides* genome assembly.

The current genome assembly of the *P. deltoides I-69* provides an essential genetic resource for mining genes underlying economically important traits and studying the evolution of the species in *Populus*. First, the new assembly provides a specific parental reference sequence for extracting tens of thousands of SNP genotypes across an F_1_ hybrid population in which the *P. deltoides I-69* was as a female parent (Mousavi et al. 2016; Tong et al. 2016). With the large number of SNPs, high-density genetic linkage maps of the parents can be constructed and thus the QTL mapping or genome-wide association studies could be conducted for growth and woody traits (Tong et al. 2020; Chen et al. 2021). Second, this genome sequence allowed us to identify genes unique to the cultivar *I-69* through orthologous analysis (Supplementary Figure 4). The unique genes may contain some detrimental genes that could cause this cultivar to have a poor rooting ability (Zhang et al. 2009). It is very important to purge these detrimental genes by selective breeding so as to develop new cultivars with improved rooting capacity for increasing the survival rate and expanding the scale of planting. Finally, the improved genome sequence would be beneficial to further elucidating the inconsistent results of phylogenetic relationships among *P. deltoides*, *P. trichocarpa*, and *P. simonii*. According to morphology, *P. trichocarpa* and *P. simonii* were assigned into the same section of Tacamahaca, while *P. deltoides* belonged to the different section of Aigeiros (Eckenwalder 1996), indicating that *P. trichocarpa* and *P. simonii* were more closely related than any other pair of the 3 species. However, recent molecular evolution analyses showed that the relationship of *P. deltoides* with *P. trichocarpa* was closer than with *P. simonii* (Zong et al. 2019; Wu et al. 2020).

## Supplementary Material

esab010_suppl_Supplementary_Data

## Data Availability

The data of Illumina short reads, 2 batches of long reads from Oxford Nanopore Technology, Hi-C reads, and RNA-seq reads have been deposited in the SRA database at the NCBI with accession numbers of SRP071167, SRR12205578, SRR12202038, SRR12975775, SRR12342994, and SRR12343732, respectively. The final genome assembly sequences and annotation file have been deposited in NCBI WGS database under the accession number GCA_015852605.1.

## References

[CIT0001] Altschul SF , Madden TL, Schäffer AA, Zhang J, Zhang Z, Miller W, Lipman DJ. 1997. Gapped BLAST and PSI-BLAST: a new generation of protein database search programs. Nucleic Acids Res. 25:3389–3402. 9254694 10.1093/nar/25.17.3389 PMC146917

[CIT0002] Bao W , Kojima KK, Kohany O. 2015. Repbase Update, a database of repetitive elements in eukaryotic genomes. Mob DNA. 6:11. 26045719 10.1186/s13100-015-0041-9 PMC4455052

[CIT0003] Biscotti MA , Olmo E, Heslop-Harrison JS. 2015. Repetitive DNA in eukaryotic genomes. Chromosome Res. 23:415–420. 26514350 10.1007/s10577-015-9499-z

[CIT0004] Burton JN , Adey A, Patwardhan RP, Qiu R, Kitzman JO, Shendure J. 2013. Chromosome-scale scaffolding of *de novo* genome assemblies based on chromatin interactions. Nat Biotechnol. 31:1119–1125. 24185095 10.1038/nbt.2727 PMC4117202

[CIT0005] Cali DS , Kim JS, Ghose S, Alkan C, Mutlu O. 2019. Nanopore sequencing technology and tools for genome assembly: computational analysis of the current state, bottlenecks and future directions. Brief Bioinform. 20:1542–1559. 29617724 10.1093/bib/bby017 PMC6781587

[CIT0006] Chen C , Chu Y, Ding C, Su X, Huang Q. 2020. Genetic diversity and population structure of black cottonwood (*Populus deltoides*) revealed using simple sequence repeat markers. BMC Genet. 21:2. 31906843 10.1186/s12863-019-0805-1 PMC6945526

[CIT0007] Chen Y , Wu H, Yang W, Zhao W, Tong C. 2021. Multivariate linear mixed model enhanced the power of identifying genome-wide association to poplar tree heights in a randomized complete block design. Genes Genomes Genetics. 11:jkaa053. 33604666 10.1093/g3journal/jkaa053 PMC8022933

[CIT0008] De Coster W , D’Hert S, Schultz DT, Cruts M, Van Broeckhoven C. 2018. NanoPack: visualizing and processing long-read sequencing data. Bioinformatics. 34:2666–2669. 29547981 10.1093/bioinformatics/bty149 PMC6061794

[CIT0009] DePristo MA , Banks E, Poplin R, Garimella KV, Maguire JR, Hartl C, Philippakis AA, del Angel G, Rivas MA, Hanna M, et al. 2011. A framework for variation discovery and genotyping using next-generation DNA sequencing data. Nat Genet. 43:491–498. 21478889 10.1038/ng.806 PMC3083463

[CIT0010] Ding X , Mei W, Lin Q, Wang H, Wang J, Peng S, Li H, Zhu J, Li W, Wang P, et al. 2020. Genome sequence of the agarwood tree *Aquilaria sinensis* (Lour.) Spreng: the first chromosome-level draft genome in the Thymelaeceae family. GigaScience. 9. doi: 10.1093/gigascience/giaa013 PMC7050300 32118265

[CIT0011] Dudchenko O , Batra SS, Omer AD, Nyquist SK, Hoeger M, Durand NC, Shamim MS, Machol I, Lander ES, Aiden AP, et al. 2017. De novo assembly of the *Aedes aegypti* genome using Hi-C yields chromosome-length scaffolds. Science. 356:92–95. 28336562 10.1126/science.aal3327 PMC5635820

[CIT0012] Dudchenko O , Shamim MS, Batra SS, Durand NC, Musial NT, Mostofa R, Pham M, Glenn St Hilaire B, Yao W, Stamenova E, et al. 2018. The Juicebox Assembly Tools module facilitates *de novo* assembly of mammalian genomes with chromosome-length scaffolds for under $1000. bioRxiv. doi: 10.1101/254797

[CIT0013] Durand NC , Robinson JT, Shamim MS, Machol I, Mesirov JP, Lander ES, Aiden EL. 2016. Juicebox provides a visualization system for Hi-C contact maps with unlimited zoom. Cell Syst. 3:99–101. 27467250 10.1016/j.cels.2015.07.012 PMC5596920

[CIT0014] Durand NC , Shamim MS, Machol I, Rao SS, Huntley MH, Lander ES, Aiden EL. 2016. Juicer provides a one-click system for analyzing loop-resolution Hi-C experiments. Cell Syst. 3:95–98. 27467249 10.1016/j.cels.2016.07.002 PMC5846465

[CIT0015] Eckenwalder JE . 1996 .Systematics and evolution of *Populus*. In: Stettler RF, Bradshaw HD, Heilman PE, Hinckley TM, editors. Biology of *Populus* and its implications for management and conservation. Ottawa (Canada): NRC Research Press, National Council of Canada. p. 7–32.

[CIT0016] Fahrenkrog AM , Neves LG, Resende MFR Jr, Dervinis C, Davenport R, Barbazuk WB, Kirst M. 2017. Population genomics of the eastern cottonwood (*Populus deltoides*). Ecol Evol. 7:9426–9440. 29187979 10.1002/ece3.3466 PMC5696417

[CIT0017] Fang L , Liu H, Wei S, Keefover-Ring K, Yin T. 2018. High-density genetic map of *Populus deltoides* constructed by using specific length amplified fragment sequencing. Tree Genet Genomes. 14:79.

[CIT0018] Gaur R , Verma S, Pradhan S, Ambreen H, Bhatia S. 2020. A high-density SNP-based linkage map using genotyping-by-sequencing and its utilization for improved genome assembly of chickpea (*Cicer arietinum* L.). Funct Integr Genomics. 20:763–773. 32856221 10.1007/s10142-020-00751-y

[CIT0019] Giani AM , Gallo GR, Gianfranceschi L, Formenti G. 2020. Long walk to genomics: history and current approaches to genome sequencing and assembly. Comput Struct Biotechnol J. 18:9–19. 31890139 10.1016/j.csbj.2019.11.002 PMC6926122

[CIT0020] Grabherr MG , Haas BJ, Yassour M, Levin JZ, Thompson DA, Amit I, Adiconis X, Fan L, Raychowdhury R, Zeng Q, et al. 2011. Full-length transcriptome assembly from RNA-Seq data without a reference genome. Nat Biotechnol. 29:644–652. 21572440 10.1038/nbt.1883 PMC3571712

[CIT0021] Guan D , McCarthy SA, Wood J, Howe K, Wang Y, Durbin R. 2020. Identifying and removing haplotypic duplication in primary genome assemblies. Bioinformatics. 36:2896–2898. 31971576 10.1093/bioinformatics/btaa025 PMC7203741

[CIT0022] Haas BJ , Delcher AL, Mount SM, Wortman JR, Smith RK Jr, Hannick LI, Maiti R, Ronning CM, Rusch DB, Town CD, et al. 2003. Improving the *Arabidopsis* genome annotation using maximal transcript alignment assemblies. Nucleic Acids Res. 31:5654–5666. 14500829 10.1093/nar/gkg770 PMC206470

[CIT0023] Haas BJ , Salzberg SL, Zhu W, Pertea M, Allen JE, Orvis J, White O, Buell CR, Wortman JR. 2008. Automated eukaryotic gene structure annotation using EVidenceModeler and the Program to Assemble Spliced Alignments. Genome Biol. 9:R7. 18190707 10.1186/gb-2008-9-1-r7 PMC2395244

[CIT0024] Han Z , Yin T, Li C, Huang M, Wu R. 2000. Host effect on genetic variation of *Marssonina brunnea* pathogenic to poplars. Theor Appl Genet. 100:614–620.

[CIT0025] Hu J , Fan J, Sun Z, Liu S. 2020. NextPolish: a fast and efficient genome polishing tool for long-read assembly. Bioinformatics. 36:2253–2255. 31778144 10.1093/bioinformatics/btz891

[CIT0026] Jiang S , An H, Xu F, Zhang X. 2020. Chromosome-level genome assembly and annotation of the loquat (*Eriobotrya japonica*) genome. GigaScience. 9. doi: 10.1093/gigascience/giaa015 PMC7059265 32141509

[CIT0027] Koren S , Walenz BP, Berlin K, Miller JR, Bergman NH, Phillippy AM. 2017. Canu: scalable and accurate long-read assembly via adaptive k-mer weighting and repeat separation. Genome Res. 27:722–736. 28298431 10.1101/gr.215087.116 PMC5411767

[CIT0028] Korf I . 2004. Gene finding in novel genomes. BMC Bioinf. 5:59. 10.1186/1471-2105-5-59 PMC421630 15144565

[CIT0029] Krzywinski M , Schein J, Birol I, Connors J, Gascoyne R, Horsman D, Jones SJ, Marra MA. 2009. Circos: an information aesthetic for comparative genomics. Genome Res. 19:1639–1645. 19541911 10.1101/gr.092759.109 PMC2752132

[CIT0030] Kurtz S , Phillippy A, Delcher AL, Smoot M, Shumway M, Antonescu C, Salzberg SL. 2004. Versatile and open software for comparing large genomes. Genome Biol. 5:R12. 14759262 10.1186/gb-2004-5-2-r12 PMC395750

[CIT0031] Lee B-Y , Park JC, Kim M-S, Choi B-S, Kim D-H, Lim J-S, Yum S, Hwang U-K, Nah GJ, Lee J-S. 2020. The genome of the Java medaka (*Oryzias javanicus*): Potential for its use in marine molecular ecotoxicology. Mar Pollut Bull. 154. doi: 10.1016/j.marpolbul.2020.111118 32319931

[CIT0032] Li H . 2013. Aligning sequence reads, clone sequences and assembly contigs with BWA-MEM. arXiv. Available from : https://arxiv.org/abs/1303.3997v2.

[CIT0033] Li H . 2018. Minimap2: pairwise alignment for nucleotide sequences. Bioinformatics. 34:3094–3100. 29750242 10.1093/bioinformatics/bty191 PMC6137996

[CIT0034] Li SF , Wang J, Dong R, Zhu HW, Lan LN, Zhang YL, Li N, Deng CL, Gao WJ. 2020. Chromosome-level genome assembly, annotation and evolutionary analysis of the ornamental plant *Asparagus setaceus*. Hortic Res. 7:48. 32257234 10.1038/s41438-020-0271-y PMC7109074

[CIT0035] Liang P , Saqib HSA, Ni X, Shen Y. 2020. Long-read sequencing and de novo genome assembly of marine medaka (*Oryzias melastigma*). BMC Genomics. 21:640. 32938378 10.1186/s12864-020-07042-7 PMC7493909

[CIT0036] Liu YJ , Wang XR, Zeng QY. 2019. *De novo* assembly of white poplar genome and genetic diversity of white poplar population in Irtysh River basin in China. Sci China Life Sci. 62:609–618. 30661181 10.1007/s11427-018-9455-2

[CIT0037] Maroso F , Hermida M, Millán A, Blanco A, Saura M, Fernández A, Dalla Rovere G, Bargelloni L, Cabaleiro S, Villanueva B, et al. 2018. Highly dense linkage maps from 31 full-sibling families of turbot (*Scophthalmus maximus*) provide insights into recombination patterns and chromosome rearrangements throughout a newly refined genome assembly. DNA Res. 25:439–450. 29897548 10.1093/dnares/dsy015 PMC6105115

[CIT0038] Michael TP , VanBuren R. 2020. Building near-complete plant genomes. Curr Opin Plant Biol. 54:26–33. 31981929 10.1016/j.pbi.2019.12.009

[CIT0039] Mousavi M , Tong C, Liu F, Tao S, Wu J, Li H, Shi J. 2016. *De novo* SNP discovery and genetic linkage mapping in poplar using restriction site associated DNA and whole-genome sequencing technologies. BMC Genomics. 17:656. 27538483 10.1186/s12864-016-3003-9 PMC4991039

[CIT0040] Oddes S , Zelig A, Kaplan N. 2018. Three invariant Hi-C interaction patterns: applications to genome assembly. Methods. 142:89–99. 29684640 10.1016/j.ymeth.2018.04.013

[CIT0041] Patel RK , Jain M. 2012. NGS QC Toolkit: a toolkit for quality control of next generation sequencing data. PLoS One. 7:e30619. 22312429 10.1371/journal.pone.0030619 PMC3270013

[CIT0042] Ran Z , Li Z, Yan X, Liao K, Kong F, Zhang L, Cao J, Zhou C, Zhu P, He S, et al. 2019. Chromosome-level genome assembly of the razor clam *Sinonovacula constricta* (Lamarck, 1818). Mol Ecol Resour. 19:1647–1658. 31483923 10.1111/1755-0998.13086

[CIT0043] Rice ES , Green RE. 2019. New approaches for genome assembly and scaffolding. Annu Rev Anim Biosci. 7:17–40. 30485757 10.1146/annurev-animal-020518-115344

[CIT0044] Roach MJ , Schmidt SA, Borneman AR. 2018. Purge Haplotigs: allelic contig reassignment for third-gen diploid genome assemblies. BMC Bioinf. 19:460. 10.1186/s12859-018-2485-7 PMC6267036 30497373

[CIT0045] Shi Y , Zhou Z, Liu B, Kong S, Chen B, Bai H, Li L, Pu F, Xu P. 2020. Construction of a high-density genetic linkage map and QTL mapping for growth-related traits in *Takifugu bimaculatus*. Mar Biotechnol (NY). 22:130–144. 31900733 10.1007/s10126-019-09938-2

[CIT0046] Simão FA , Waterhouse RM, Ioannidis P, Kriventseva EV, Zdobnov EM. 2015. BUSCO: assessing genome assembly and annotation completeness with single-copy orthologs. Bioinformatics. 31:3210–3212. 26059717 10.1093/bioinformatics/btv351

[CIT0047] Slater GS , Birney E. 2005. Automated generation of heuristics for biological sequence comparison. BMC Bioinf. 6:31. 10.1186/1471-2105-6-31 PMC553969 15713233

[CIT0048] Stanke M , Keller O, Gunduz I, Hayes A, Waack S, Morgenstern B. 2006. AUGUSTUS: *ab initio* prediction of alternative transcripts. Nucleic Acids Res. 34:W435–W439. 16845043 10.1093/nar/gkl200 PMC1538822

[CIT0049] Tarailo-Graovac M , Chen N. 2009. Using RepeatMasker to identify repetitive elements in genomic sequences. Curr Protoc Bioinformatics. 25:4.10.1–14.10.14. 10.1002/0471250953.bi0410s25 19274634

[CIT0050] Tarasov A , Vilella AJ, Cuppen E, Nijman IJ, Prins P. 2015. Sambamba: fast processing of NGS alignment formats. Bioinformatics. 31:2032–2034. 25697820 10.1093/bioinformatics/btv098 PMC4765878

[CIT0051] Ter-Hovhannisyan V , Lomsadze A, Chernoff YO, Borodovsky M. 2008. Gene prediction in novel fungal genomes using an *ab initio* algorithm with unsupervised training. Genome Res. 18:1979–1990. 18757608 10.1101/gr.081612.108 PMC2593577

[CIT0052] Tong C , Li H, Wang Y, Li X, Ou J, Wang D, Xu H, Ma C, Lang X, Liu G, et al. 2016. Construction of high-density linkage maps of *Populus deltoides x P. simonii* using restriction-site associated DNA sequencing. PLoS One. 11:e0150692. 26964097 10.1371/journal.pone.0150692 PMC4786213

[CIT0053] Tong C , Yao D, Wu H, Chen Y, Yang W, Zhao W. 2020. High-quality SNP linkage maps improved QTL mapping and genome assembly in *populus*. J Hered. 111:515–530. 32930789 10.1093/jhered/esaa039 PMC7751148

[CIT0054] Tuskan GA , Difazio S, Jansson S, Bohlmann J, Grigoriev I, Hellsten U, Putnam N, Ralph S, Rombauts S, Salamov A, et al. 2006. The genome of black cottonwood, *Populus trichocarpa* (Torr. & Gray). Science. 313:1596–1604. 16973872 10.1126/science.1128691

[CIT0055] Vaser R , Sović I, Nagarajan N, Šikić M. 2017. Fast and accurate de novo genome assembly from long uncorrected reads. Genome Res. 27:737–746. 28100585 10.1101/gr.214270.116 PMC5411768

[CIT0056] Wei S , Yang Y, Yin T. 2020. The chromosome-scale assembly of the willow genome provides insight into Salicaceae genome evolution. Hortic Res. 7:45. 32257231 10.1038/s41438-020-0268-6 PMC7109076

[CIT0057] Wu H , Yao D, Chen Y, Yang W, Zhao W, Gao H, Tong C. 2020. *De novo* genome assembly of *Populus simonii* further supports that *Populus simonii* and *Populus trichocarpa* belong to different sections. G3 (Bethesda). 10:455–466. 31806765 10.1534/g3.119.400913 PMC7003099

[CIT0058] Xing Y , Liu Y, Zhang Q, Nie X, Sun Y, Zhang Z, Li H, Fang K, Wang G, Huang H, et al. 2019. Hybrid de novo genome assembly of Chinese chestnut (*Castanea mollissima*). GigaScience. 8. doi: 10.1093/gigascience/giz112 PMC6741814 31513707

[CIT0059] Xu Z , Dixon JR. 2020. Genome reconstruction and haplotype phasing using chromosome conformation capture methodologies. Brief Funct Genomics. 19:139–150. 31875884 10.1093/bfgp/elz026 PMC7334751

[CIT0060] Yang W , Wang K, Zhang J, Ma J, Liu J, Ma T. 2017. The draft genome sequence of a desert tree *Populus pruinosa*. GigaScience. 6:1–7. 10.1093/gigascience/gix075 PMC5603765 28938721

[CIT0061] Zhang B , Tong C, Yin T, Zhang X, Zhuge Q, Huang M, Wang M, Wu R. 2009. Detection of quantitative trait loci influencing growth trajectories of adventitious roots in *Populus* using functional mapping. Tree Genet Genomes. 5:539–552.

[CIT0062] Zhang Z , Chen Y, Zhang J, Ma X, Li Y, Li M, Wang D, Kang M, Wu H, Yang Y, et al. 2020. Improved genome assembly provides new insights into genome evolution in a desert poplar (*Populus euphratica*). Mol Ecol Resour. 20:781–794. 10.1111/1755-0998.13142 32034885

[CIT0063] Zhu S , Cao YZ, Jiang C, Tan BY, Wang Z, Feng S, Zhang L, Su XH, Brejova B, Vinar T, et al. 2012. Sequencing the genome of *Marssonina brunnea* reveals fungus-poplar co-evolution. BMC Genomics. 13:382. 22876864 10.1186/1471-2164-13-382 PMC3484023

[CIT0064] Zong D , Gan P, Zhou A, Zhang Y, Zou X, Duan A, Song Y, He C. 2019. Plastome sequences help to resolve deep-level relationships of *Populus* in the Family Salicaceae. Front Plant Sci. 10:5. 30723484 10.3389/fpls.2019.00005 PMC6349946

