# Supplementary material for: Genome Assembly of Salicaceae Populus deltoides (Eastern Cottonwood) I-69 Based on Nanopore Sequencing and Hi-C Technologies
Source: J Hered. 2021 Mar 17;112(3):303–10. doi: 10.1093/jhered/esab010 (PMC8141683; doi:10.1093/jhered/esab010)
Supplement: esab010_suppl_Supplementary_Data [file esab010_suppl_supplementary_data.docx]

**Supplementary Tables and Figures**

**Supplementary Table 1** Summary for the chromosomal assembly of *Populus deltoides* based on Nanopore sequencing and Hi-C data.

**Supplementary Table 2** Comparison in chromosome sizes among the genomes of *P. deltoides*, *P. euphratica*, *P. trichocarpa* and *P. simonii*.

**Supplementary Table 3** Summary statistics of interspersed repeats.

**Supplementary Table 4** Summary statistics of gene prediction results.

**Supplementary Figure 1** Length distribution of Nanopore long reads.

**Supplementary Figure 2** Read-depth analysis for removing heterozygous sequences. All the Nanopore long reads were mapped onto the polished contigs and the sequencing depth of all the positions in the genome was plotted. A bimodal distribution was observed that the left peak (~50x) representing for haploid level of coverage, whereas the right peak (~100x) representing for diploid level of coverage.

**Supplementary Figure 3** Circos plot showing macro-synteny between the genomes of *P. deltoides I-69* and *P. deltoides WV94*. Each connecting line represents a syntenic fragment of 10 Kb.

**Supplementary Figure 4** OrthoVenn diagram of the orthologous groups from the genomes of *P. deltoides*, *P. euphratica*, *P. trichocarpa*, *P. simonii* and *S. suchowensis*.

**Table S1** Summary for the chromosomal assembly of *Populus deltoides* based on Nanopore sequencing and Hi-C data.

| Chromosomes | Sequence Length (bp) | Contig Number |
| --- | --- | --- |
| Chr01 | 53167185 | 94 |
| Chr02 | 26326550 | 33 |
| Chr03 | 25925424 | 74 |
| Chr04 | 23785796 | 34 |
| Chr05 | 25191203 | 32 |
| Chr06 | 27008206 | 20 |
| Chr07 | 16499584 | 20 |
| Chr08 | 20706301 | 38 |
| Chr09 | 13452252 | 28 |
| Chr10 | 22314285 | 24 |
| Chr11 | 21385715 | 64 |
| Chr12 | 15516283 | 27 |
| Chr13 | 17879110 | 42 |
| Chr14 | 19275714 | 31 |
| Chr15 | 15442390 | 16 |
| Chr16 | 16692328 | 32 |
| Chr17 | 18142355 | 51 |
| Chr18 | 17774561 | 38 |
| Chr19 | 21510302 | 68 |
| Total | 417995544 | 766 |

**Supplementary Table 2** Comparison in chromosome sizes among the genomes of *P. deltoides*, *P. euphratica*, *P. trichocarpa* and *P. simonii*.

|  |  | Sequence Length (bp) | | |
| --- | --- | --- | --- | --- |
|  | *P. deltoides* | *P. euphratica* | *P. trichocarpa* | *P. simonii* |
| Chr01 | 53,167,185 | 63,758,542 | 49,788,581 | 52,212,564 |
| Chr02 | 26,326,550 | 28,821,247 | 25,242,375 | 24,814,715 |
| Chr03 | 25,925,424 | 28,590,778 | 21,678,634 | 23,004,272 |
| Chr04 | 23,785,796 | 41,241,862 | 24,140,038 | 22,273,817 |
| Chr05 | 25,191,203 | 36,707,947 | 24,981,103 | 25,305,605 |
| Chr06 | 27,008,206 | 33,417,250 | 27,516,652 | 28,474,677 |
| Chr07 | 16,499,584 | 26,919,898 | 15,561,420 | 17,173,342 |
| Chr08 | 20,706,301 | 25,947,872 | 19,195,260 | 17,033,540 |
| Chr09 | 13,452,252 | 21,781,469 | 12,987,399 | 16,172,202 |
| Chr10 | 22,314,285 | 36,152,031 | 22,799,081 | 23,004,089 |
| Chr11 | 21,385,715 | 22,409,682 | 19,288,771 | 19,598,675 |
| Chr12 | 15,516,283 | 22,096,726 | 15,589,050 | 15,715,777 |
| Chr13 | 17,879,110 | 21,495,510 | 15,705,617 | 16,467,678 |
| Chr14 | 19,275,714 | 21,686,686 | 17,801,709 | 18,415,095 |
| Chr15 | 15,442,390 | 21,175,825 | 15,231,745 | 14,201,725 |
| Chr16 | 16,692,328 | 19,092,525 | 14,619,816 | 15,138,618 |
| Chr17 | 18,142,355 | 30,671,117 | 15,189,755 | 15,010,321 |
| Chr18 | 17,774,561 | 27,927,787 | 16,264,003 | 14,400,979 |
| Chr19 | 21,510,302 | 19,863,256 | 15,623,655 | 19,936,707 |
| Total | 417,995,544 | 549,758,010 | 389,204,664 | 398,354,398 |

**Table S3** Summary statistics of interspersed repeats.

| Type | Number of elements | Length occupied (bp) | Percentage of sequence (%) |
| --- | --- | --- | --- |
| Retroelements | 141265 | 101582894 | 23.66 |
| SINEs: | 16253 | 2550300 | 0.59 |
| Penelope | 163 | 97760 | 0.02 |
| LINEs: | 8866 | 4567936 | 1.06 |
| CRE/SLACS | 1 | 64 | 0.00 |
| L2/CR1/Rex | 570 | 152214 | 0.04 |
| R1/LOA/Jockey | 54 | 9999 | 0.00 |
| RTE/Bov-B | 394 | 83976 | 0.02 |
| L1/CIN4 | 7681 | 4223806 | 0.98 |
| LTR elements: | 116146 | 94464658 | 22.00 |
| BEL/Pao | 93 | 27535 | 0.01 |
| Ty1/Copia | 36379 | 25699433 | 5.99 |
| Gypsy/DIRS1 | 77452 | 67467908 | 15.71 |
| Retroviral | 260 | 147843 | 0.03 |
|  |  |  |  |
| DNA transposons | 62913 | 29326912 | 6.83 |
| hobo-Activator | 17418 | 6120503 | 1.43 |
| Tc1-IS630-Pogo | 453 | 105649 | 0.02 |
| En-Spm | 0 | 0 | 0.00 |
| MuDR-IS905 | 0 | 0 | 0.00 |
| PiggyBac | 0 | 0 | 0.00 |
| Tourist/Harbinger | 8949 | 4306723 | 1.00 |
| Other (Mirage, P-element, Transib) | 0 | 0 | 0.00 |
|  |  |  |  |
| Unclassified: | 157253 | 37742887 | 8.79 |
|  |  |  |  |
| Total interspersed repeats: | 361431 | 168652693 | 39.28 |

**Table S4** Summary statistics of gene prediction results.

| Type |  |
| --- | --- |
| Number of genes | 32,245 |
| Number of CDSs | 44,362 |
| Average number of exons per CDS | 5.6 |
| Mean exon length | 306 bp |
| Mean intron length | 421 bp |
| Mean CDS length | 1,258 bp |
| Mean gene length | 3,527 bp |

**
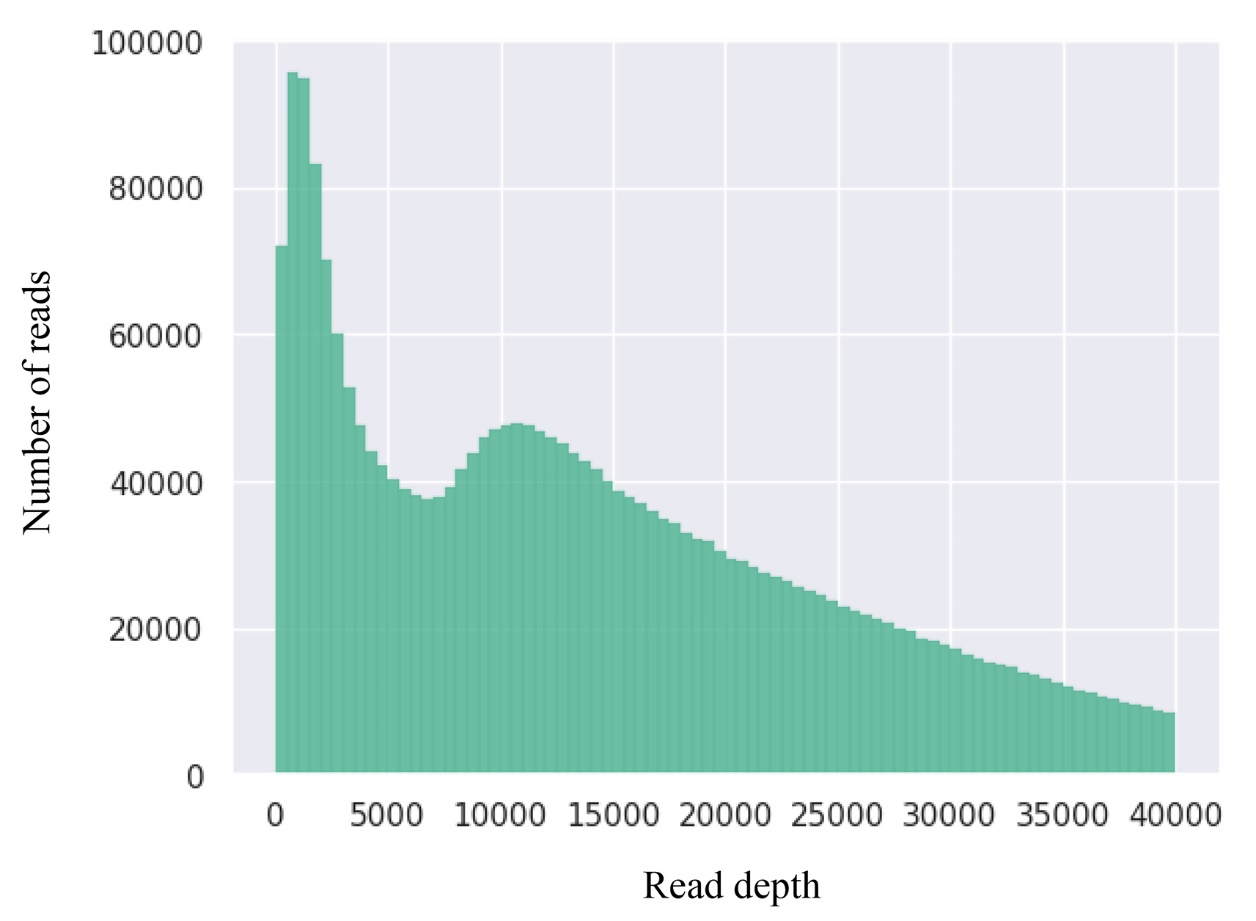
**

**Figure S1** Length distribution of Nanopore long reads.


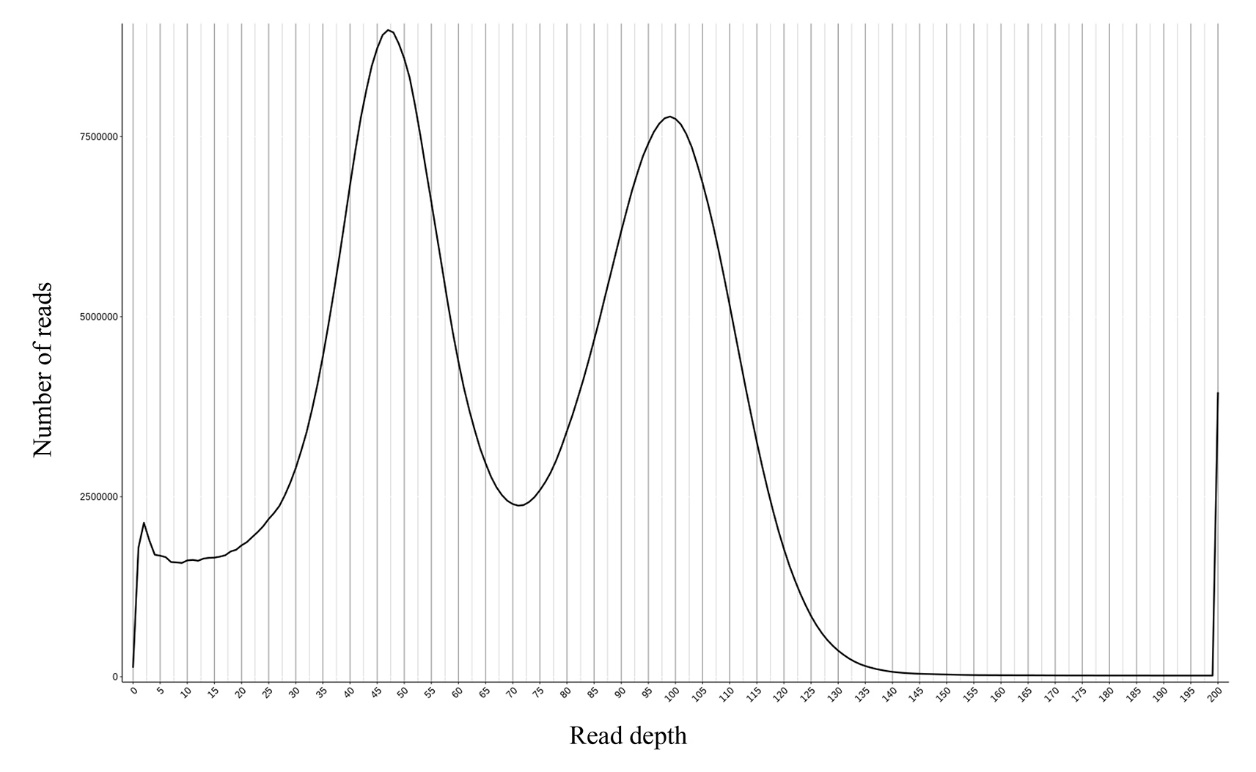


**Figure S2** Read-depth analysis for removing heterozygous sequences. All the Nanopore long reads were mapped onto the polished contigs and the sequencing depth of all the positions in the genome was plotted. A bimodal distribution was observed that the left peak (~50x) representing for haploid level of coverage, whereas the right peak (~100x) representing for diploid level of coverage.

**
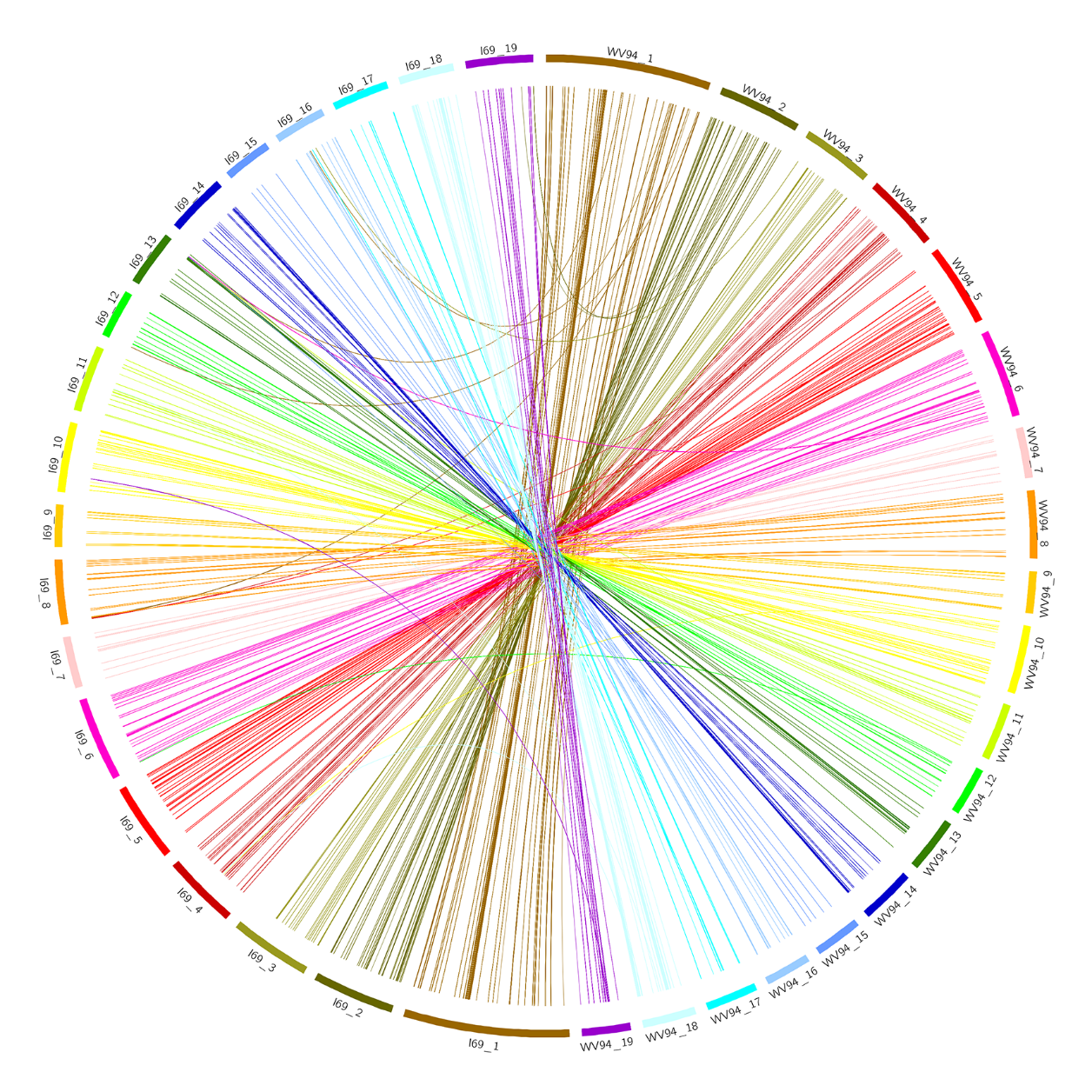
**

**Supplementary Figure 3** Circos plot showing macro-synteny between the genomes of *P. deltoides I-69* and *P. deltoides WV94*. Each connecting line represents a syntenic fragment of 10 Kb.


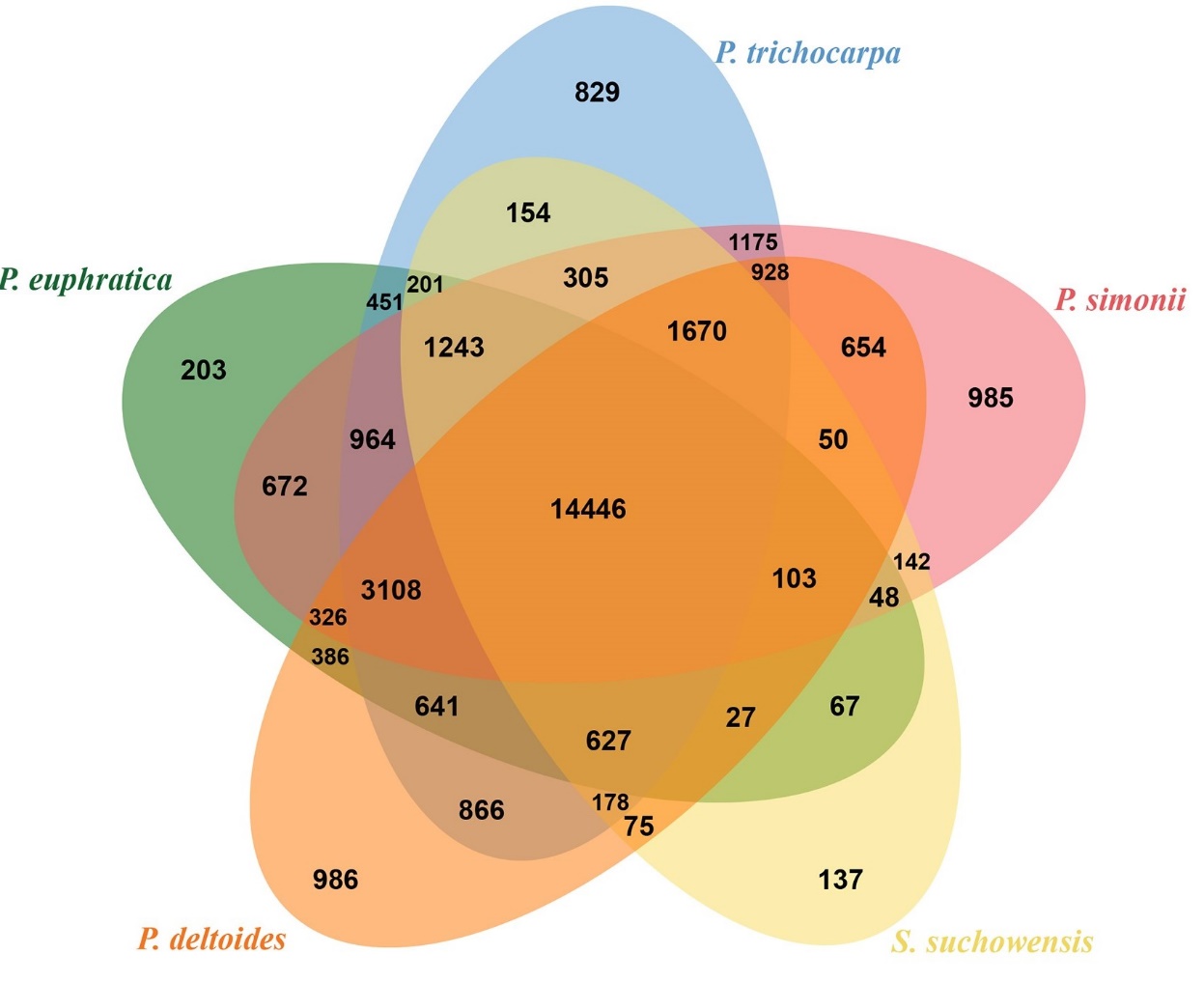


**Supplementary Figure 4** OrthoVenn diagram of the orthologous groups from the genomes of *P. deltoides*, *P. euphratica*, *P. trichocarpa*, *P. simonii* and *S. suchowensis*.
